# Reduced continuity index with proactive esophageal cooling compared to luminal temperature monitoring during radiofrequency ablation

**DOI:** 10.1016/j.hroo.2025.02.010

**Published:** 2025-02-21

**Authors:** Catherine Lazarus, Jacob Sherman, Natalie Putzel, Cameron Randolph, William Zagrodzky, Tiffany Sharkoski, Alex Ro, Jose Nazari, Westby Fisher, Erik Kulstad, Mark D. Metzl

**Affiliations:** 1 Northwestern University, Evanston, Illinois; 2 Washington University in St. Louis, St. Louis, Missouri; 3 University of Southern California, Los Angeles, California; 4 Dr. Kiran C. Patel College of Osteopathic Medicine, Clearwater, Florida; 5 Attune Medical, now a part of Haemonetics, Boston, Massachusetts; 6 Endeavor Health, Evanston, Illinois; 7 University of Texas Southwestern Medical Center, Dallas, Texas

**Keywords:** Proactive esophageal cooling, Atrial fibrillation, Lesion contiguity, Continuity index, Long-term freedom from atrial fibrillation, Pulmonary vein isolation, Radiofrequency ablation

## Abstract

**Background:**

Proactive esophageal cooling is Food and Drug Administration (FDA) cleared to reduce the likelihood of esophageal injury during radiofrequency ablation for treatment of atrial fibrillation (AF). Long-term follow-up data have also shown improved freedom from arrhythmia with proactive esophageal cooling compared with luminal esophageal temperature (LET) monitoring during pulmonary vein isolation (PVI). One hypothesized mechanism is improved lesion contiguity (as measured by the continuity index) with the use of cooling.

**Objective:**

We aimed to compare the continuity index of PVI cases using proactive esophageal cooling with those using LET monitoring.

**Methods:**

We calculated the continuity index for PVI cases at 2 different hospitals within the same health system, using a slightly modified continuity index to facilitate retrospective determination from review of recorded cases. The results were then compared between cases using proactive esophageal cooling and those using LET monitoring.

**Results:**

Continuity Indices for a total of 236 cases were determined: 118 cases using proactive esophageal cooling and 118 cases using traditional LET monitoring. With proactive esophageal cooling, the average continuity index was 10.6 (5.6 on the left pulmonary vein and 4.9 on the right pulmonary vein). With LET monitoring, the average continuity index was 37.0 (18.7 on the left and 18.3 on the right), for a difference of 26.4 (*P* < .001).

**Conclusion:**

Proactive esophageal cooling during PVI is associated with significantly improved lesion contiguity when compared with LET monitoring. This finding may offer a mechanism for the greater freedom from arrhythmia seen with proactive cooling in long-term follow-up.

**Key Findings**

▪ Data suggest improved long-term freedom from arrhythmia when lesion contiguity is optimized during pulmonary vein isolation.
  ○ Proactive esophageal cooling during pulmonary vein isolation has been associated with improved long-term freedom from arrhythmia when compared with luminal esophageal temperature (LET) monitoring.
▪ This study compared lesion contiguity between LET monitoring and proactive esophageal cooling, finding that lesion contiguity, as measured by a continuity index, was significantly improved with cooling.
▪ These findings are likely attributable to esophageal cooling’s role in eliminating the need to pause and reposition the ablation catheter in response to esophageal temperature increases.

## Introduction

Atrial fibrillation (AF) is a common form of cardiac arrhythmia increasingly treated with radiofrequency (RF) pulmonary vein isolation (PVI) ablation.^1^ As new technologies continue to emerge, improving procedural efficacy while maintaining procedural safety remains paramount. Procedural efficacy, as measured by long-term freedom from arrhythmia, has been shown to increase with improved contiguity of lesion placement.^2^^,^^3^ However, lesion contiguity is often limited by the need to pause and reposition the ablation catheter in response to temperature increases in the esophagus when using luminal esophageal temperature (LET) monitoring. Temperature increases in the esophagus generally require cessation of RF delivery and result in delays in placing the adjacent lesion while awaiting return to equilibrium temperatures or performing repositioning of the catheter elsewhere in the left atrium.

An increasingly used alternative to LET monitoring is proactive esophageal cooling using a dedicated esophageal cooling device. Proactive esophageal cooling during RF ablation has been shown to reduce endoscopically identified esophageal lesions by 83%,^4^ and a recent analysis of 25,186 patients found a significant reduction in atrioesophageal fistulas with cooling.^5^ This approach is now also cleared by the US Food and Drug Administration (FDA) to reduce the likelihood of ablation-related esophageal injury resulting from RF cardiac ablation procedures.^6^

In addition to enhanced safety, recent data have shown improved long-term freedom from arrhythmia with proactive esophageal cooling when compared with LET monitoring during PVI. The long-term follow-up from the 120-patient IMPACT randomized controlled trial found a 3% (*P* = NS) absolute improvement in freedom from arrhythmia at 12 months.^7^ A larger 513-patient review (from which the patients described in this study were obtained) found a 14% absolute improvement (*P* = .03) in freedom from arrhythmia at 1 year.^8^

A potential mechanism for an improvement in long-term freedom from AF with proactive esophageal cooling is via improvement in lesion contiguity, or lesion sequentiality.^3^^,^^9^ Lesion contiguity has been characterized using the continuity index, which is a quantification determined by the number of noncontiguous, or nonadjacent, lesions placed.^3^ Each noncontiguous lesion increments the continuity index, such that the higher the continuity index, the lower the overall contiguity of lesions. Cases with lower continuity indices have been shown to result in better isolation and greater freedom from arrhythmia when compared with procedures with higher continuity indices.^3^ Likewise, cases with higher continuity indices exhibit higher rates of gap reformation because of the rapid edema formation that occurs immediately after lesion placement, preventing subsequent lesions from achieving full-thickness transmurality.^3^ Proactive esophageal cooling eliminates the need to pause RF lesion placement because of temperature rises in the esophagus, because no temperature monitoring is used with the system. As such, proactive esophageal cooling may allow the continuity index to be lower than is otherwise possible when using LET monitoring. To test this hypothesis, we measured the continuity index of PVI cases and compared the results between cases using proactive esophageal cooling and cases using LET monitoring.

## Materials and methods

### Study design

This institutional review board–approved study included a retrospective review of PVI cases using RF ablation performed by 4 operators. Investigators calculated the continuity index by reviewing case recordings from the 3-D mapping system (Carto, Biosense Webster, Inc., Diamond Bar, CA) used during PVI cases.

### Patients

Patients included those undergoing PVI procedures for the treatment of AF by any of 4 operators. AF types included paroxysmal, persistent, and long-standing persistent AF.

### Data collection

The methods for calculating the continuity index in real time have been described previously,^10^ but in brief, investigators reviewed recordings of cases contained in the 3-D mapping system and calculated the continuity index for each case while also recording patient age and sex. Further details of the calculation are as described in the following paragraphs.

### Procedure

RF PVI ablations were performed similarly to those of most procedures performed in the United States, with patients under general anesthesia, and wide area circumferential PVI performed with the use of electroanatomic and 3-dimensional geometry mapping using the Carto 3 mapping system and an irrigated ablation ST/SF™ catheter (Biosense Webster, Inc., Diamond Bar, CA). Contact force catheters were used in all cases. Mitral isthmus lines, additional posterior wall isolation, and cavotricuspid isthmus lines were employed dependent on physician preference. A high-power short-duration technique was targeted, and low- or no-fluoroscopy approach was used, as described in a previous publication.^11^ The pulmonary veins were isolated by delivery of RF applications circumferentially to the antral regions of the veins to produce a minimum of entrance and exit block for at least 20 minutes. A Smartablate™ generator (Biosense Webster) was used to deliver RF energy, with a setpoint of 50 W in all cases and in all areas of the left atrium. The Visitag Surpoint™ (Biosense Webster) module (ablation index) was used during ablations, with a target of 400 units on the posterior wall, and 550 units on the anterior wall, lateral wall, and septum. An intertag distance of less than 6 mm was targeted. The Visitag sizes were all 3-mm-radius lesions. Before March 2019, LET monitoring was used, whereas after March 2019, proactive esophageal cooling was used. There were no other differences in the procedural approach besides the type of esophageal protection. All operators involved in this study had more than 8 years of experience at the beginning of the time frame analyzed.

**Figure 1:**
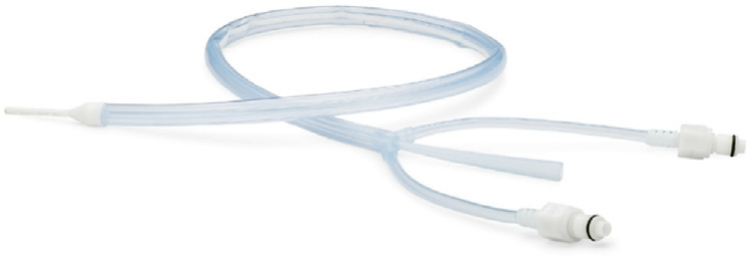
Proactive esophageal cooling device.

In the cases in which proactive esophageal cooling was employed, a dedicated cooling device (ensoETM®, Attune Medical, Chicago, IL) consisting of a closed-loop multi-lumen medical-grade silicone tube that circulates cold water at a temperature of 4°C was placed (Figure 1). The device circulates distilled water at a rate of 2.4 L/minute between the device and an external heat exchanger. The device replaces the need for LET monitoring and is positioned similarly to an orogastric tube, avoiding interference with procedural workflow. Device placement is confirmed by either fluoroscopy or intracardiac echocardiography.

In the cases using LET monitoring, a multi-sensor probe (Circa S-Cath; Circa Scientific, Inc., Englewood, CO) was employed. Energy delivery was discontinued when the maximum LET on any sensor of the multi-sensor probe rose by more than 0.2°C/s or exceeded 39°C.

### Continuity index calculation

**Figure 2:**
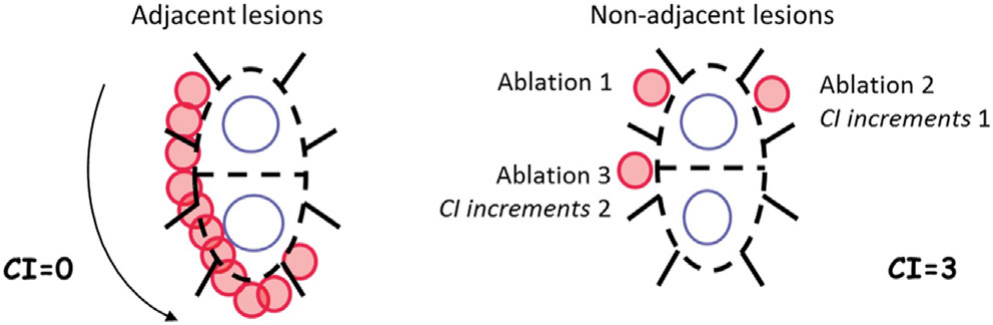
Original continuity index^3^ (reprinted with permission).

The original description of the Continuity Index divided the left atrium into segments, and incremented the Index by the number of segments the catheter was moved to place a noncontiguous lesion (Figure 2).^3^

**Figure 3:**
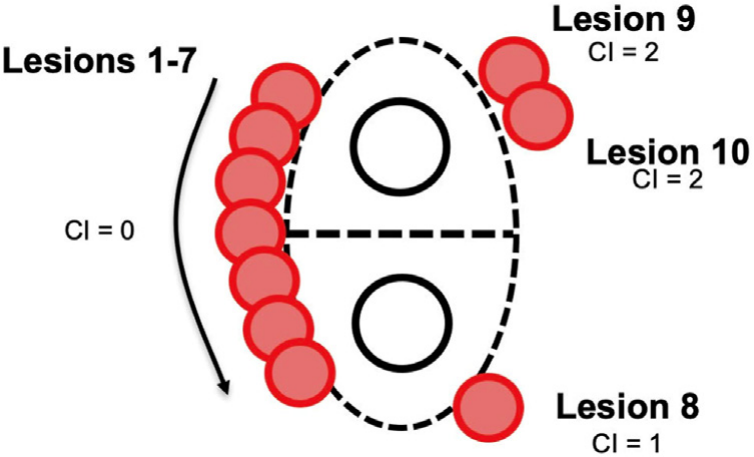
Minor modification to the continuity index (CI). “Lesions 1–7” represent a continuity index of 0, because each lesion is directly adjacent or overlapping with the previous lesion. The deployment of “lesion 8” makes the continuity index 1, because there is a discontinuity between lesions 7 and 8 with no direct overlap. Similarly, “lesion 9” further increased the continuity index to 2 because it is not contiguous with “lesion 8.” There is no change with the addition of “lesion 10,” because it is directly adjacent to “lesion 9.”

To better facilitate standardization and improve ease of use as applied to recorded cases, we used a slight modification, incrementing the Index by 1 unit for each lesion that does not border a previous lesion, regardless of distance placed from the previous lesion (Figure 3). This modification avoided the ambiguity inherent with estimating exact demarcations of segments of the left atrium, which otherwise would add variability and increase subjectivity in determination of the continuity index. As an example, if an operator deployed every lesion adjacent to the previous lesion without any discontinuity, the final continuity index would be 0. Each lesion placed noncontiguously would increment the index by 1 unit.

### Data analysis

The mean continuity index was calculated for each group, and results compared between the LET group and the esophageal cooling group. Data were analyzed using SPSS (IBM SPSS Statistics for Windows, Version 29.0., IBM Corp, Armonk, NY) Continuous variables are presented as mean ± SD.

## Results

### Patients

The Continuity Index was determined for a total of 236 cases. Of these, 118 using proactive esophageal cooling were obtained for cases performed from March 2019 to November 2019. Another 118 cases using LET monitoring (before adoption of proactive esophageal cooling, which occurred in March 2019) were determined.

### Patient characteristics

Patient demographics for both treatment groups were similar. The average age for the proactively cooled group was 69.4 (standard deviation [SD], ±9.4) years, and the average age was 65.81 (SD, ±12.2) years for the LET monitored group. Likewise, there was no significant difference in sex between the 2 groups, with a greater percentage male in each, and the proactively cooled group consisting of 60.2% male and the LET monitored group consisting of 72.9% male (*P* = NS). Table 1 provides further characteristics of patients representing a larger group of over 500 patients, from which the subset reported here were drawn.

**Table 1:** Further characteristics of patients representing a larger group of over 500 patients, from which the subset reported here were drawn

Age	68.35 ± 10.23
Female sex	211 (37.4)
Left atrial diameter, mm	43.02 ± 7.05
LVEF, %	56.86 ± 12.66
Paroxysmal AF	301 (53.4)
Hx BB	378 (64.0)
Hx CCB	91 (16.1)
Hx AADs	212 (37.6)
Hypertension	245
Diabetes mellitus	129 (22.9)
Heart failure	69 (12.2)
CHA2DS2 VASC	2.46 ± 1.55

AAD = antiarrhythmic drug; AF = atrial fibrillation; BB = beta blocker; CCB = calcium channel blocker; Hx = history; LVEF = left ventricular ejection fraction.

### Continuity index comparison

**Figure 4:**
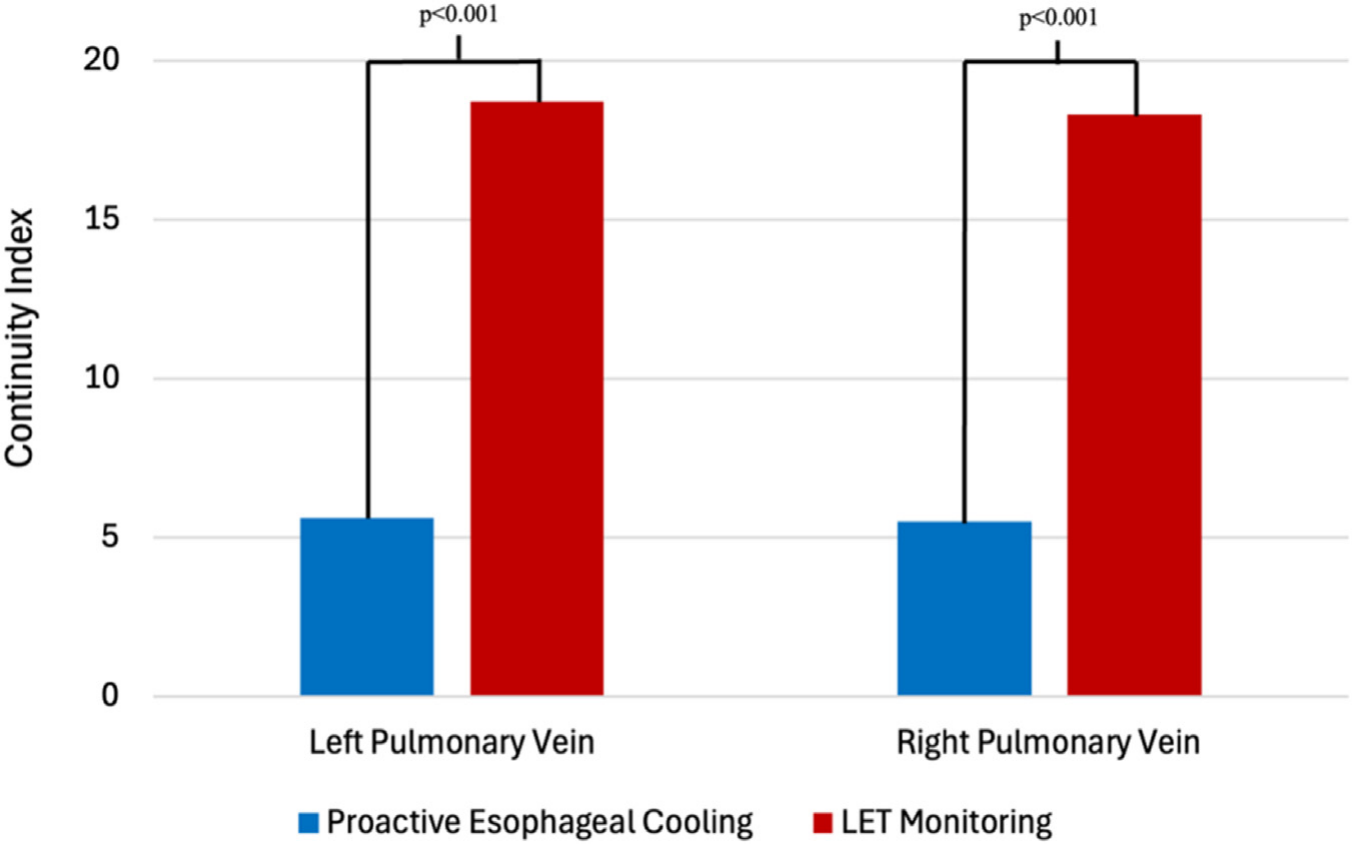
Comparison of continuity index (modified) between LET monitoring and proactive esophageal cooling.

Cases that used proactive esophageal cooling had a mean continuity index of 4.9 (SD, ±5.4) for the right pulmonary veins and 5.6 (SD, ±5.5) for the left pulmonary veins, for a mean total continuity index of 10.6 (SD, ±10.2) (Table 2). Cases using LET monitoring had a mean continuity index of 18.3 (SD, ±6.59) for the right pulmonary veins and 18.7 (SD, ±6.4) for the left pulmonary veins, for a mean total continuity index of 37.0 (SD, ±11.4), representing a significant order of magnitude difference (*P* < .001) (Figure 4). As reported in a prior publication, at 1-year follow-up, Kaplan-Meier estimates for patients in the cohort from which these continuity indices were obtained found that freedom from AF was 58.2% for LET-monitored patients and 72.2% for actively cooled patients.^8^ Procedure time differences between LET monitoring and esophageal cooling have been previously reported to be 36 minutes shorter with esophageal cooling, attributed to the avoidance of the need to pause lesion placement or reposition the RF catheter because of overheating.^11^

**Table 2:** Detailed continuity index parameters

	Continuity index		
	Cooling (n = 118)	LET monitoring (n = 118)	
Left side			
Mean	5.6	18.7	*P* < .001
SD	5.5	6.4	
Min	0	7	
Max	27	37	
Right side			
Mean	4.9	18.3	*P* < .001
SD	5.4	6.5	
Min	0	6	
Max	24	40	
Total			
Mean	10.6	37.0	*P* < .001
SD	10.2	11.4	
Min	0	15	
Max	49	73

## Discussion

This is the first study to compare the continuity index obtained with different methods of esophageal protection, finding that the continuity index is significantly lower when using proactive esophageal cooling than with LET monitoring. Cases using proactive esophageal cooling had a mean continuity index of 10.6, whereas those using LET monitoring had a mean continuity index of 37.0 (*P* < .001). Proactive esophageal cooling enabled significant improvement in lesion contiguity by eliminating interruptions of RF delivery that would otherwise be encountered in cases employing LET monitoring.

The formal measure of contiguity via a continuity index was proposed by Kautzner et al^3^ in the EFFICAS-II study in 2015.^3^ In this study, continuity indices above 6 (signifying a lower lesion contiguity) corresponded to a significantly diminished likelihood of preserving long-term freedom from AF when compared with cases with continuity indices below 6 (signifying a higher degree of lesion contiguity).^3^ Specifically, PV lines isolated initially with a continuity index below 6 had a 98% (56/57) chance of remaining isolated, as compared with PV lines with a continuity index ≥ 6, which had only a 62% (21/34) chance of remaining isolated (*P* < .001). Further methods of quantifying lesion sequentiality have since been described by Jankelson et al,^9^ who found that arrhythmia-free survival was significantly higher in patients with greater lesion sequentiality (96% vs 76%; *P* = .01).^9^ The higher occurrence of gaps is attributable to at least 2 factors. The first is the partial transmurality that results from premature cessation of RF energy after detecting an intraesophageal temperature rise. The second is the fact that edema begins to form around each lesion soon after lesion placement.^12^, ^13^, ^14^ This growing edema hinders subsequent transmural lesion formation at adjacent positions, because once the operator returns to the site to place an adjacent lesion, the tissue is no longer the same thickness and will require different energy parameters to achieve transmurality.^2^ In cases with LET monitoring, it is common practice to reposition the ablation catheter to other regions of the atrium to prevent heat stacking and overheating.^15^

The temporal aspect of lesion placement is important, because lesions placed immediately are more likely to exhibit transmurality associated unipolar electrograms as evidence of lesion transmurality.^2^ In cases with lower continuity indices, there is less time for edema to form around lesions, providing a higher likelihood of achieving transmurality because of more consistent tissue thickness.^3^ In proactively cooled cases, there is no compulsion to interrupt RF delivery, because the esophagus is proactively cooled to 4°C, and no temperature monitoring is required. This approach additionally offers greater safety, because, in contrast to LET monitoring, which has not demonstrated benefit in reducing esophageal injury,^16^, ^17^, ^18^ proactive esophageal cooling has demonstrated significant reduction in both esophageal injury and atrioesophageal fistula rates.^4^^,^^5^ As such, LET monitoring remains unapproved for esophageal protection by the FDA, whereas proactive esophageal cooling is FDA cleared to reduce the likelihood of ablation-related esophageal injury resulting from RF cardiac ablation procedures.^6^

### Study limitations

Given the nature of this study, investigators were not blinded to the mode of esophageal protection used. However, objective endpoints of lesion placement order are unlikely to be influenced by knowledge of mode of esophageal protection. Cases with LET monitoring were performed by 3 of the 4 operators using proactive esophageal cooling, but this study otherwise followed the same operators with consistent procedural approaches, operating within the same hospital system, and serving the same patient demographic over the time period. Although only a subset of a larger data set, long-term follow-up comparison of procedural outcome for the cases reviewed in this analysis are described in an earlier paper.^8^ Prospective randomized controlled trial data^7^ also support the improved efficacy from proactive esophageal cooling; however, direct linkage of lesion contiguity to arrhythmia recurrence rate will require additional prospective study, because the subset here were drawn from the Carto system, in which detailed patient characteristics are not retained. Although procedure times are not reported in this study, multiple studies have shown improved procedure time reductions of 10% to 40% when using esophageal cooling as opposed to LET monitoring.^19^, ^20^, ^21^, ^22^, ^23^

## Conclusion

Proactive esophageal cooling during PVI is associated with significantly improved lesion contiguity when compared with LET monitoring. This finding may offer a mechanism for the greater freedom from arrhythmia seen with proactive cooling in long-term follow-up.

## Disclosures

Catherine Lazarus, Jacob Sherman, Natalie Putzel, Cameron Randolph, and William Zagrodzky have interned with Attune Medical; Tiffany Sharkoski has employment with Haemonetics; Erik Kulstad has consulted for Haemonetics; Mark Metzl has consulted for Abbott, Biosense Webster, Haemonetics, Medtronic, Sanofi Aventis and Phillips. Alex Ro, Jose Nazari and Westby Fisher have no disclosures.
